# Nuclear destabilisation – a possible genesis of cancer?

**DOI:** 10.1111/brv.70052

**Published:** 2025-07-14

**Authors:** Daniel D. Scott, Francesco Bettariga, Marco Ventin, Chris Bishop, Britta Stordal

**Affiliations:** ^1^ London Sport Institute, Faculty of Science & Technology Middlesex University London NW4 4BT UK; ^2^ Department of Natural Sciences, Faculty of Science & Technology Middlesex University London NW4 4BT UK; ^3^ Exercise Medicine Research Institute, Edith Cowan University Prilep Drive Joondalup Western Australia 6027 Australia; ^4^ School of Medical and Health Sciences Edith Cowan University Prilep Drive Joondalup Western Australia 6027 Australia; ^5^ Department of Surgery Cedars Sinai Medical Center Los Angeles California 90048 USA

**Keywords:** cancer, cell mechanics, nucleosome, chromatin, cell destabilisation, mechanical genesis

## Abstract

This review examines the increasingly prominent role of mechanics within cancer formation and progression. The extremely varied and contradictory genetic landscape of cancer is in stark contrast to the seemingly universal mechanical characteristics of cancer cells and their tumour microenvironment, and mechanics may be a principal unifying trait of this disease. The tight regulation of innate cell mechanical properties raises the possibility that destabilisation of the cell drives tumour formation in an attempt to restore cell mechanical homeostasis. With losses in cell stiffness more pronounced at the cell nucleus, we hypothesise that destabilisation occurs within the nucleus, likely within the nucleosome. Beyond the mechanical properties of the cell, this compromise to the chromatin structure holds significant repercussions for both genetic and epigenetic regulation, providing scope for significant genetic dysregulation and mutation. However, the nature of such genetic events will be dependent upon the region of mechanical destabilisation; thus, introducing greater variability and heterogeneity to genetic changes. We conclude with the hypothesis that cancer has a mechanical genesis, in which cell nuclear destabilisation functions as the enabling hallmark of cancer. It is theorised that both genetic and structural dysfunction stem from this nuclear destabilisation, driving disease pathology and progression.

## I. INTRODUCTION

Cancer is a major health burden worldwide, with nearly 20 million new cases and 9.7 million deaths estimated in 2022 alone (Bray *et al*., 2024). The rate of cancer incidence is expected to reach over 35 million new cases per year in 2050, corresponding to a 77% increase from the 20 million estimated cases in 2022 (Bray *et al*., 2024). Despite significant progress in determining contributing mechanisms and pathways, the origins and causes of these pathways still remain unclear (Hanahan & Weinberg, 2011). This leaves the diverse and pervasive characteristics of cancer only partially understood, with the ultimate genesis of cancer requiring further research (Erenpreisa *et al*., 2014; Farahmund *et al*., 2023; Hanselmann & Welter, 2016; Paduch, 2015). Cancer possesses multiple characteristic traits, including changes to cell stiffness (Daniel *et al*., 2023; Fischer, Hayn & Mierke, 2020; Guck *et al*., 2005; Lin *et al*., 2015; Massey *et al*., 2024; Xu *et al*., 2012), increased proliferation (Sun *et al*., 2018; Sun, Pardo & Kumar, 2009), altered genetic sequences (Knouse *et al*., 2017; Taylor *et al*., 2018), apoptosis resistance (Neophytou *et al*., 2021; Todaro *et al*., 2008), immune evasion (Kim & Cho, 2022; Thelen *et al*., 2021; Yoshihama *et al*., 2016), altered cell signalling (Gu *et al*., 2012; Hutz *et al*., 2017), and tumour formation (Zhang *et al*., 2021a; Zhang *et al*., 2021b). This highlights that cancer is dependent upon the interaction of genetic, chemical, and mechanical pathways, resulting in a highly complex and interrelated landscape.

Although cancer is often considered from a genetics perspective (Brucher & Jamali, 2016; Sonnenschein & Soto, 2000; Paduch, 2015), mechanical considerations have been receiving increasing attention in recent years (Broders‐Bondon *et al*., 2018; Cheng *et al*., 2009; Ishihara & Haga, 2022; Reid *et al*., 2017; Sonneschein & Soto, 2000; Yu *et al*., 2022). Fundamentally, these mechanical investigations examine alterations to stress and strain, and how these alterations interact with cell physiology (Kumar & Weaver, 2009; Reid *et al*., 2017; Tian, Lin & Zhang, 2021). This provides a working definition within this review, with “mechanics” defined as the interaction of mechanical load and its resultant physiological stress and strain. The importance of mechanics within cancer is exemplified at both tissue and cell levels, with changes to both associated with increased invasiveness and aggressiveness of cancers (Fischer *et al*., 2020; Reid *et al*., 2017). At the tissue level, stiffness of the tumour microenvironment is increased (Reid *et al*., 2017; Tian *et al*., 2021), a mechanical change possibly preceding cancer formation (Ishihara & Haga, 2022; Martinez & Smith, 2021). By contrast, the stiffness of cancer cells is decreased (Daniel *et al*., 2023; Fischer *et al*., 2020; Guck *et al*., 2005; Lin *et al*., 2015; Massey *et al*., 2024; Xu *et al*., 2012), a phenomenon evident at the cell cytoskeleton and nucleus (Daniel *et al*., 2023; Massey *et al*., 2024). These characteristics create a possibly universal mechanical property of cancer, in which cells decrease in stiffness whilst the surrounding tissue stiffness increases. Thus, understanding of these mechanical signatures of cancer may provide insight into cancer, possibly from its earliest stages, and thereby aid in developing new treatments and improving treatment efficacy. These findings can be understood as the latest progress in work dating back to the mid‐19th century (e.g. Beale, 1860). Such work observed that cancer cells had nuclear morphology alterations, greater nucleoli numbers, multinucleation, and an increased nuclear size relative to the whole cell size (Singh & Lele, 2022). Indeed, changes in nuclear morphology have been used as a diagnostic tool from their discovery through to modern times (Abel *et al*., 2024; Singh & Lele, 2022; Sengupta *et al*., 2022). Thus, mechanics can be understood to be intimately linked with cancer pathology, with this association recognised in early research in cancer (Beale, 1860). Ongoing work on the mechanics of cancer continues to expand upon these contributions within the cancer pathology research space.

Although mechanics is receiving growing interest for its contributions to our understanding of cancer progression (Broders‐Bondon *et al*., 2018; Cheng *et al*., 2009; Ishihara & Haga, 2022; Reid *et al*., 2017; Sonneschein & Soto, 2000; Yu *et al*., 2022), the origins of these mechanical events are poorly understood. Due to this limited understanding, we only have an incomplete picture of the full implications and role of mechanics within cancer. Therefore, the purpose of this review is to provide an overview on the loss of nuclear stiffness during cancer formation and progression, and detail how it can contribute to several critical components of cancer pathology. We suggest that destabilisation of the cell nucleus is the genesis of cancer, with this mechanical catalyst triggering genetic and structural cascades that ultimately produce the cancer cell.

## II. MECHANICS AND CELL DEVELOPMENT AND FUNCTION

The cell is intimately tied to its mechanical environment and the forces acting upon it. Mechanics informs cell functions such as differentiation and cell fate (Engler *et al*., 2004; Huang, Dai & Zhang, 2015; Putra, Killian & Tate, 2023; Vining & Mooney, 2017; Zhang, Zhang & Wang, 2022), growth (Wang *et al*., 2020), migration (Gjorevski *et al*., 2015; Plutoni *et al*., 2016; Zeng *et al*., 2012), and transcription (Alam *et al*., 2016; Di *et al*., 2023; McCubrey *et al*., 2007). Thus, mechanics is significant in the origins of any differentiated cell and continues to guide and mediate function and form throughout the cell lifespan. Even during mitosis (Martin & Cardoso, 2010) and cell death (Rose *et al*., 2022), mechanics is a significant contributor. This tight interplay between mechanics and all facets of cell function stands in stark relief to the dysregulated mechanical state of cancer cells. The reduced stiffness of cancer cells (Daniel *et al*., 2023; Fischer *et al*., 2020; Guck *et al*., 2005; Lin *et al*., 2015; Massey *et al*., 2024; Swaminathan *et al*., 2011; Xu *et al*., 2012) is accompanied by compromised or altered mechano‐sensing (Yang *et al*., 2020). Therefore, understanding the interplay of different mechanical cues in healthy cells can inform how the behaviour of cancer cells is changed and compromised by these mechanical alterations.

The initial transformation of stem cells into tissue‐specific cells (cell differentiation) is heavily mediated by the mechanical properties of the substrate the cells are attached to (Engler *et al*., 2004, 2006; Huang *et al*., 2015). Using an *in vitro* hydrogel method, Engler *et al*. (2006) compared the stiffness of three different substrates of 0.1–1.0, 8–17, and 25–40 kPa, to simulate brain, muscle, and bone extracellular matrix environments, respectively. Using mesenchymal stem cells (MSCs), Engler *et al*. (2006) demonstrated that extracellular matrix stiffness altered cell differentiation. The softest substrate stiffness, mimicking brain tissue, resulted in MSCs becoming primary neuron‐like cells. The moderate stiffness, matching striated muscle, produced spindle‐shaped cells consistent with myoblasts. Finally, the stiffest substrate equivalent to bone tissue produced osteoblast‐like cells. Additionally, when mechano‐sensing by MSCs was compromised, morphological changes were inhibited. Thus, the mechanical environment is a crucial component of differentiation, altering cell structure, mechanical properties, and transcription (Engler *et al*., 2006). These findings have been extensively replicated throughout the literature (Cao *et al*., 2022; Charrier *et al*., 2018; Mullen *et al*., 2015; Olivares‐Navarrete *et al*., 2010, 2017; Park *et al*., 2011).

The results of Engler *et al*. (2006) help further to contextualise this mechanical interplay between cell and tissue and demonstrated that as substrate stiffness increased, cell prestress increased, creating higher cell stiffness. Additionally, administration of Blebbistatin (a non‐muscle myosin II inhibitor) compromised morphological changes and suppressed key lineage markers, ultimately inhibiting differentiation. They also found that as substrate stiffness increased, so too did focal adhesion size and F‐actin organisation. Focal adhesions link the cell cytoskeleton to the surrounding extracellular matrix, and are a critical component in translating external mechanical cues to cellular outcomes (Seong, Wang & Wang, 2013; Sigaut *et al*., 2018), a process known as mechano‐transduction (Chen, 2008; Di *et al*., 2023). Focal adhesions are formed, in part, by non‐muscle myosin II, F‐actin, and integrins (Legerstee & Houtsmuller, 2021). They are dynamic structures able to form and dismantle rapidly, a process regulated by non‐muscle myosin II (Pasapera *et al*., 2010; Stricker *et al*., 2013). Non‐muscle myosin II directs changes to the cell cytoskeleton, directing F‐actin towards focal adhesions (Chan *et al*., 2015; Lehtimaki *et al*., 2021; Shutova *et al*., 2012), whilst F‐actin alignment is directed by the enacting forces (Griener *et al*., 2013; Li *et al*., 2020). Therefore, mechanosensitive non‐muscle myosin II (Schiffhauer *et al*., 2019) probes the mechanical environment and reorganises the cell structure accordingly, producing the variable adhesion size and F‐actin organisation, noted in prior research (Engler *et al*., 2006). The apparent mechanical isolation and inhibition of differentiation by blocking non‐muscle myosin II availability observed by Engler *et al*. (2006) demonstrates that re‐organisation of the cell structure and its mechanical properties is a necessary component of mechanically derived cell function and behaviour. Thus, these results support cell mechanical properties as a fundamental component of cell function.

The ability of mechanics to influence the cell is strongly mediated by interplay with cell genetics and epigenetics. Engler *et al*. (2006) provided evidence of this, demonstrating that transcription factors [proteins that up‐ or down‐regulate specific genes (Latchman, 1993; Stadhouders, Filion & Graf, 2019)] and transcriptomes are specific to the extracellular matrix stiffness. Park *et al*. (2011) utilised an *in vitro* gel study using MSCs, and found that extracellular matrix stiffness mediated specific gene expression, including lineage‐specific expression, supporting a role for mechanical regulation of gene expression in cell differentiation. Olivares‐Navarrete *et al*. (2017) provided supporting evidence from an *in vitro* gel study utilising MSCs and osteoblasts. They found that regulation of the RUNX2 transcription factor was dependent on extracellular matrix stiffness in MSCs, but only varied at the softest substrate stiffness in osteoblasts. Importantly, their results suggested that on softer substrates, the genetic expression levels of osteoblasts became so low as to suggest de‐differentiation. Osteoblasts differ mechanically from MSCs, including in stiffness (Darling *et al*., 2008), with this differing mechanical profile producing different gene expression outcomes. Cumulatively, these findings indicate that mechanical mediation of cell genetics is dependent upon the cells mechanical profile itself. This again supports internal cell mechanics specifically as a central mediator of mechanically derived cell behaviour. However, there is further regulation through the interplay of cell type and extracellular matrix stiffness, as data indicate that the extracellular matrix mediates the morphology of cells and their gene transcription (Engler *et al*., 2006; Olivares‐Navarette *et al*., 2017). This extends beyond stem cells alone, as Olivares‐Navarette *et al*. (2017) suggest that sufficient change to the mechanical environment can lead to de‐differentiation. With differentiation describing the process of stem cell commitment to a specific cell type, this de‐differentiation describes the loss of specific cell markers and a return to more stem cell‐like markers. Such findings indicate that the maintenance of the mechanical environment mediates the retention of differentiation. Therefore, the stability of a cell's behaviour may be partially, yet meaningfully, determined by the stability of the mechanical environment. Thus, results indicate that cell mechanical properties are a fundamental driver of cell functions, with these mechanical properties themselves mediated by extracellular matrix stiffness, consistent with the symbiotic perspective of cell and extracellular matrix described in mechano‐transduction (Di *et al*., 2023).

Although the symbiotic relationship between cell and extracellular matrix is consistent with mechano‐transduction, the central role of innate cell mechanical properties may suggest limitations to the classic mechano‐transduction description of mechano‐regulated cell function. The mechano‐transduction process is well characterised by findings showing that strain of three‐dimensional microtissues results in strain‐softening of the cells (Walker *et al*., 2020), with strain‐softening functioning to preserve absolute cell stiffness as the cell undergoes strain. These data clearly detail external forces acting upon cells and initiating a response. However, within this, the potential limitations of mechano‐transduction are also illustrated. The cell strain‐softening response occurs to preserve the absolute mechanical properties of the cell, yet mechano‐transduction does not consider the effects of altered cell mechanics independent from external forces. Therefore, the results provide a new perspective, in which cells maintain a stiffness homeostasis, with mechano‐transduction working by disturbing this homeostasis. Arising from this, the internal mechanical state supersedes external mechanical cues, and all mechanically derived cell actions are the sum effect of all mechanical forces and the deviation from homeostasis they create. This has important implications when considering cancer.

## III. ALTERED MECHANICS IN CANCER

### (1) Cell mechanics and its relationship to the surrounding tissue

The mechanical environments within cancer exhibit several critical changes compared with healthy cells and tissue. These changes can be broadly categorised as altered cell mechanics (Daniel *et al*., 2023; Fischer *et al*., 2020; Massey *et al*., 2024), altered tissue mechanics (Massey *et al*., 2024; Paszek *et al*., 2005), and altered cell mechano‐sensing (Yang *et al*., 2020). As discussed in Section II, extracellular matrix stiffness directs cell properties and thus differentiation, ultimately having broad effects on cell function and behaviour. However, within cancer, alterations to cell and tissue properties indicate this relationship is bi‐directional. Metastatic potential increases as cell stiffness reduces (Fischer *et al*., 2020; Wullkopf *et al*., 2018; Young *et al*., 2023), demonstrating that losses to cell stiffness are an important factor in cancer progression. However, in contrast to healthy cells, this change in cell mechanical profile appears somewhat independent of the surrounding extracellular matrix properties. Breast and pancreatic cancer cells seeded in collagen I extracellular matrix maintain their diminished stiffness despite increasing extracellular matrix stiffness (Wullkopf *et al*., 2018). Indeed, within mammary tissue, tumour tissue is around 24 times stiffer (mean ± SEM 167 ± 31 *versus* 4049 ± 938 Pa; Paszek *et al*., 2005), yet cancer cells maintain their compromised mechanical profile (Massey *et al*., 2024). It should be noted that increased extracellular matrix stiffness can lead to marginal increases in cancer cell stiffness, although not universally and overall stiffness remains significantly below healthy cell values (Wullkopf *et al*., 2018). However, the tissue stiffness did not reach levels consistent with tumour stiffness (Massey *et al*., 2024). Therefore, it is possible that cancer cell mechanics are augmented within the tumour microenvironment.

**Fig. 1:**
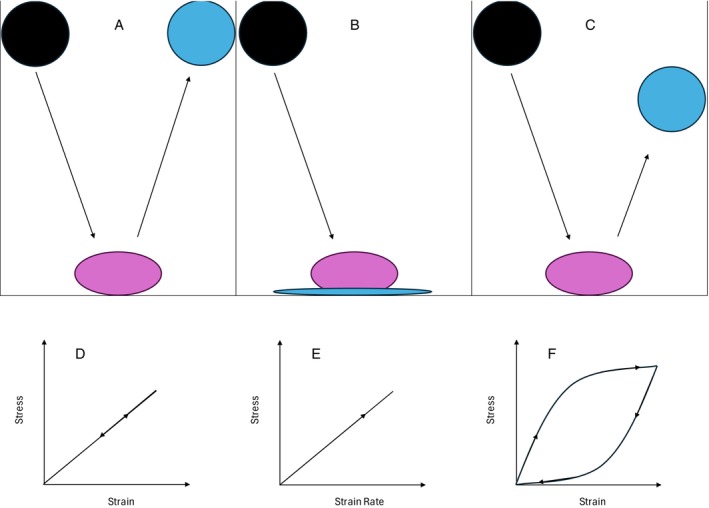
Illustration of the characteristics of pure elastic (A), viscous (B), and viscoelastic (C) materials. Black balls show the starting condition, pink the condition at impact, and blue the condition after impact, with black arrows showing the direction of travel. Example stress and strain profiles are given in the graphs below: elastic (D), viscous (E), and viscoelastic (F). Perfectly elastic materials (A and D) demonstrate zero energy loss alongside a proportional relationship between stress and strain. By contrast, viscous materials dissipate energy, typically as heat. This dissipation typically leads to permanent changes in structure. Additionally, unlike elastic materials, viscous materials do not have a proportional relation between stress and strain. Rather stress is proportional to the rate of strain, as depicted in E. Finally, viscoelastic materials (C and F) regain their initial shape when stress is removed, showing elastic behaviours. However, dissipation of energy whilst under strain and mechanical properties that vary depending upon the rate of strain (Özkaya & Nordin, 1999) are viscous behaviours. This combination of elastic and viscous behaviours gives rise to the stress/strain loop (F), known as hysteresis, which details the loss of energy during strain (Özkaya & Nordin, 1999).

Understanding this altered mechanical relationship between the cell and extracellular matrix may have significant implications for cancer research and treatment. Using an *in vitro* single‐cell experiment, Tian *et al*. (2020) compared cell viscoelasticity (see Fig. 1) and focal adhesion tension in non‐tumorigenic (MCF‐10A) and metastatic (MDA‐MB‐231) breast cells on different polyacrylamide gel substrate stiffnesses. There were four magnitudes of stiffness of substrate used: mean ± SD 1.0 ± 0.1, 5.9 ± 0.4, 12.8 ± 0.7, and 89.4 ± 6.7 kPa. Results showed that non‐tumorigenic cells varied little in viscoelasticity and focal adhesion tension across all stiffnesses except for at 12.8 kPa where both measures showed a slight percentage increase. By contrast, the malignant cell type demonstrated increasing focal adhesion tension and viscoelasticity as substrate stiffness increased. The results of Engler *et al*. (2006) indicate that focal adhesions directly modulate cell prestress and thus can mediate whole‐cell stiffness. Furthermore, greater extracellular matrix stiffness produces larger focal adhesions (Pelham & Wang, 1997), generating greater traction forces and thereby further augmenting cell prestress (Engler *et al*., 2006). Thus, increasing extracellular matrix stiffness allows greater augmentation of cell stiffness (Tian *et al*., 2020). Additionally, cell elastic or viscoelastic behaviour is heavily mediated by the type of cytoskeleton crosslinking, with more transient and non‐covalently bonded crosslinks increasing dermal fibroblast cell viscoelasticity (Burla *et al*., 2019; Chaubet *et al*., 2020). Thus, the results of Tian *et al*. (2020) evidence a less‐stable cell structure that depends on extracellular matrix adhesions to combat decreased cell stiffness and mechanical stability. Additionally, Hu *et al*. (2018) examined non‐tumorigenic (MCF‐10A), metastatic (MDA‐MB‐468), and highly metastatic (MDA‐MB‐231) breast cancer cells. Results demonstrated that increasing metastatic ability was accompanied by decreased polymerised actin content, with MDA‐MB‐231 cells displaying a 75% reduction compared to MCF‐10A. The redistribution of the majority of actin to the cell periphery in cancer cells (Hu *et al*., 2018), alongside actin organisation based on enacting forces (Griener *et al*., 2013; Li *et al*., 2020), all suggest the predominant forces acting on the cell are from focal adhesions. Additionally, the reduction in total actin and its redistribution to the cell periphery (Hu *et al*., 2018) are consistent with a loss in cell stiffness/prestress (Daniel *et al*., 2023; Massey *et al*., 2024). Further, in cancer cells, actin loss is consistent with increased capacity for mechanical insulation from external cues (Yang *et al*., 2020), as actin remodelling and the transfer of force along actin are critical components within cell mechano‐sensing (Blanchoin *et al*., 2014; Li *et al*., 2020).

These findings show that increases to tissue stiffness are not independent of cell mechanical changes but are a direct result of the loss in cell stiffness. Therefore, the tumour microenvironment can be categorised as an adaptive process, creating a structural scaffold to stabilise the cell. Important evidence in support of this conclusion is provided by Cirka *et al*. (2016), who used a custom stretch system and compliant well structure system to investigate porcine valvular interstitial cell (VIC) and human osteosarcoma cell (U20S) traction forces to long‐term stretch. Manipulation of cell prestress was achieved through pre‐treatment of VICs with transforming growth factor‐β1 (TGF‐β1) or Blebbistatin to increase or decrease prestress respectively. TGF‐β1 has been shown to increase VIC prestress by augmenting contractile forces of the cell (Cushing, Liao & Anseth, 2005). By contrast, Blebbistatin decreases cell stiffness by inhibiting non‐muscle myosin II, hindering cell prestress. They demonstrated that cell traction forces and contractile moments increased in cells with low prestress yet decreased in cells with increased prestress. This supports innate cell mechanical properties regulating the interactions between extracellular matrix and cell. The increasing stretch within this study is relevant to the tumour microenvironment, as increasing stretch equates to increased stiffness (Cirka *et al*., 2016; Engler *et al*., 2006). Therefore, the results suggest that within the tumour microenvironment, healthy cells can maintain their mechanical profile whilst cancerous cells can augment their failing structural integrity. Thus, the tumour microenvironment creates selective mechanical support, only affecting the cancerous cells. This is likely explained by healthy cells under strain modulating prestress to preserve absolute stress (Walker *et al*., 2020; Tian *et al*., 2020; Cirka *et al*., 2016).

A second relevant finding from Cirka *et al*. (2016) was that Blebbistatin‐treated VICs showed outward displacement at the cell periphery. The authors suggested this may be due to polymerisation of actin stress fibres and/or non‐recoverable stretch of stress fibres. However, bulging protrusions from within the cell membrane, known as blebbing (Fackler & Grosse, 2008; Fang *et al*., 2017), provide further insight. Blebbing is caused by disassociation of the cell membrane and the actin cytoskeleton (Fackler & Grosse, 2008; Fang *et al*., 2017). Fluids within the cells are restrained by the cytoskeleton, and thus weakening of the cytoskeleton can allow fluid pressures to create blebs, or extrusions from the main cell body. The use of Blebbistatin by Cirka *et al*. (2016) affects the structure of the cell periphery, as non‐muscle myosin II mediates the peripheral cytoskeleton structure and regulates prestress (Chan *et al*., 2015; Lehtimaki *et al*., 2021; Shutova *et al*., 2012). Thus, the outward spreading at the cell edge observed in the Blebbistatin‐treated VICs group may indicate that the hydrostatic pressure was beginning to exceed the cytoskeletal stiffness. This is consistent with observations in cancer cells as blebbing has been associated with increased metastasis of prostate (PC3, DU145) (Khan *et al*., 2019), human fibrosarcoma (HT‐1080), breast carcinoma (MDA‐MB‐231, MDA‐MB‐435), colon adenocarcinoma (HT‐29), lung carcinoma (BZR), hepatocarcinoma (HUH‐7, PLC‐PRF‐5) (Weems *et al*., 2023), and melanoma (SK‐MEL‐28, HT‐144, A375, MV3, M498) (Brassart *et al*., 2019; Weems *et al*., 2023) cells. Khan *et al*. (2019) attributed blebbing to losses in actin cytoskeleton, and this suggests blebbing within cancer cells is consistent with typical cell blebbing, in which fluid pressure from the cytoplasm exceeds the structural capacity of the cell cytoskeleton. Furthermore, data show that blebs have the capacity to function as extracellular matrix adhesion sites (Guzman *et al*., 2020). Within rounded breast cancer cells (MDA‐MB‐231 and MDA‐MB‐468), blebs were found to adhere to, and reorganise, collagen fibres within the extracellular environment. Thus, blebs (which indicate a compromise in cell structural integrity), contain the ability to augment the cell mechanical properties. The frequent observation of blebs within cancer cells (Guzman *et al*., 2020) indicates both a compromise to structure, and a dynamic response to improve cell stiffness through extracellular matrix adhesions. This evidence further supports the failing mechanical state of the cell as a potent driver of cancer, but also indicates mechanical similarity between the Blebbistatin‐treated cells of Cirka *et al*. (2016) and the diminished prestress of cancer cells. Placing the results of Cirka *et al*. (2016) within the broader findings detailed herein, they support the tumour functioning as a selective scaffold, reinforcing structurally failing cells by augmenting innate cell mechanical properties. This adaptive process indicates that these innate cell mechanical properties supersede external mechanical cues and suggests the loss in cell stiffness is a primary concern within cancer. This mechanical loss would seem concentrated at the nucleus. Ordinarily, the nucleus is 2–10 times stiffer than the cytoskeleton (Caille, Tardy & Meister, 1998; Caille *et al*., 2002; Guilak, Tedrow & Burgkart, 2000), yet in cancerous cells this difference between nucleus and cytoskeleton stiffness is significantly reduced to only 1–1.5 times larger (Fischer *et al*., 2020; Massey *et al*., 2024), suggesting the loss of nuclear stiffness may be the important change.

In contrast to other cancer cell types, chondrosarcoma has been reported to exhibit increased cell stiffness (Daniel *et al*., 2023). This increased stiffness directly challenges the mechanical perspective presented herein. Unfortunately, mechanical investigations of chondrosarcoma cells are limited, and these investigations rarely include detail of the nuclear structure and its mechanics. As actin content is reduced, consistent with other cancers, the increased cytoskeleton stiffness was attributed to increased β‐tubulin expression and content. β‐tubulin is a component of the cell cytoskeleton (Verma *et al*., 2023), forming microtubules in conjunction with α‐tubulin (Zhang *et al*., 2016). The structural contribution of microtubules within the cytoskeleton is resistance to compression (Brangwynne *et al*., 2006). Using atomic force microscopy, Daniel *et al*. (2023) illustrated this structural role of microtubules, as their assessment of cytoskeletal stiffness utilised compression. Given this, the increased cytoskeletal stiffness in chondrosarcoma may be specific to compression only, whilst the loss of actin suggests a potential reduction in tensional stiffness (Li *et al*., 2020). Providing further context to the variance in cytoskeleton components, Verma *et al*. (2023) examined the effects of substrate stiffness on cytoskeleton proteins within several cell lines (HMF3S, HT1080 & CCL‐64 PAI). Using silicone elastomer substrates mixed with different ratios of cross‐linkers Verma *et al*. (2023) demonstrated that decreasing substrate stiffness resulted in losses of actin but increases in β‐tubulin, mirroring the results of Daniel *et al*. (2023). Thus, the data from Verma *et al*. (2023) may indicate that their extracellular matrix environment (Leibovitz's L‐15 medium without L‐glutamine) is more compliant than the cartilage environment chondrosarcomas originate from. However, a critical component of the Daniel *et al*. (2023) study is the use of the corresponding healthy cell as a control. The increased β‐tubulin within chondrosarcoma cells was determined relative to healthy chondrocyte cells within the same extracellular matrix environment. Thus, Daniel *et al*. (2023) demonstrate an increase in cytoskeleton stiffness under compression. However, the cytoskeleton protein concentrations indicate alterations in stiffness may be specific to a given type of force, with compression increased whilst tension is decreased. This lack of certainty in the condition of the cytoskeleton provides a challenge in determining the implications of the results of Daniel *et al*. (2023). Despite this, the data demonstrate that mechanical changes are observed between healthy and cancerous cells independent of the extracellular matrix environment, reinforcing the suggestion that changes in cellular mechanics originate from within the cell, and are not dependent on changes to the extracellular matrix environment. Furthermore, the data suggest that losses in nuclear stiffness are more pronounced than in the cytoskeleton of cancer cells (Fischer *et al*., 2020; Massey *et al*., 2024) and the evidence presented for cell internal mechanical properties as the ultimate arbiter of mechanically derived cell behaviour (e.g. Walker *et al*., 2020) focus attention away from the cytoskeleton and onto the nucleus.

Unfortunately, Daniel *et al*. (2023) did not investigate the nucleus, however, Tan, Choong & Dass (2010) conducted a somewhat limited investigation of the chondrosarcoma nucleus following treatment with pigment epithelium‐derived factor (PEDF), which is known to impede cancer through angiogenesis inhibition (Becerra & Notario, 2013). Compared to untreated control cells, PEDF‐treated cells had increased apoptosis concurrent with increased chromatin density. Results are consistent with the presence of decondensed chromatin within chondrosarcoma nuclei, which would incur reductions in nuclear stiffness (Nava *et al*., 2020). Supporting this, a report of eight case studies (Jakowski & Wakely, 2007) found chromatin to be evenly distributed within rounded nuclei. With rounded nuclei indicative of reduced tensional forces (Nyga *et al*., 2023) and a lack of condensed chromatin suggesting reduced nuclear stiffness (Nava *et al*., 2020), the results are consistent with a compromised nuclear structure. In conjunction with the other findings described above, there thus seems tentative support for compromised nuclear mechanics despite the presence of increased cytoskeleton stiffness. Although further research is necessary, this is consistent with the general hypothesis presented herein, that nuclear mechanics is the critical consideration.

### (2) Cell nuclear mechanics

The nucleus is the largest organelle within a cell, housing and regulating genetic and epigenetic materials and functions (Hertzog & Erdel, 2023). Its structure is dominated by lamins and chromatin (Dechat, Adam & Goldman, 2009; Stephens, Banigan & Marko, 2017; Xu *et al*., 2022). Lamins A and C (A/C) are encoded by the *LMNA* gene (Lin & Worman, 1993) and create a boundary perimeter at the nucleus periphery (Adam, 2017; Xu *et al*., 2022). They are a primary component of the nucleoskeleton (Adam, 2017; Crisp & Burke, 2008) and are an integral link between the nucleoskeleton and cytoskeleton (LINC) complexes (Adam, 2017). Thus, lamins A/C are important within the transmission of mechanical forces across the cell and mediate mechanical integration between cell periphery and components within the nucleus, such as chromatin (Kim *et al*., 2017; Wang *et al*., 2022; Xu *et al*., 2022). B‐type lamins also contribute to the nucleoskeleton mechanical properties (Nmezi *et al*., 2019). However, there is debate over the viability of cells lacking B‐type lamins. In HeLa cells, it was found that RNA interference (RNAi)‐mediated knockdown of lamin B1 or B2 arrested growth and resulted in apoptosis (Harborth *et al*., 2001). Other studies have shown that some cell types can function with altered B‐type lamins, although this may be limited to specific cell types (Yang *et al*., 2011). We therefore limit our discussion here to lamins A/C, as their contributions appear more universal across different cell types (Xu *et al*., 2022).

**Fig. 2:**
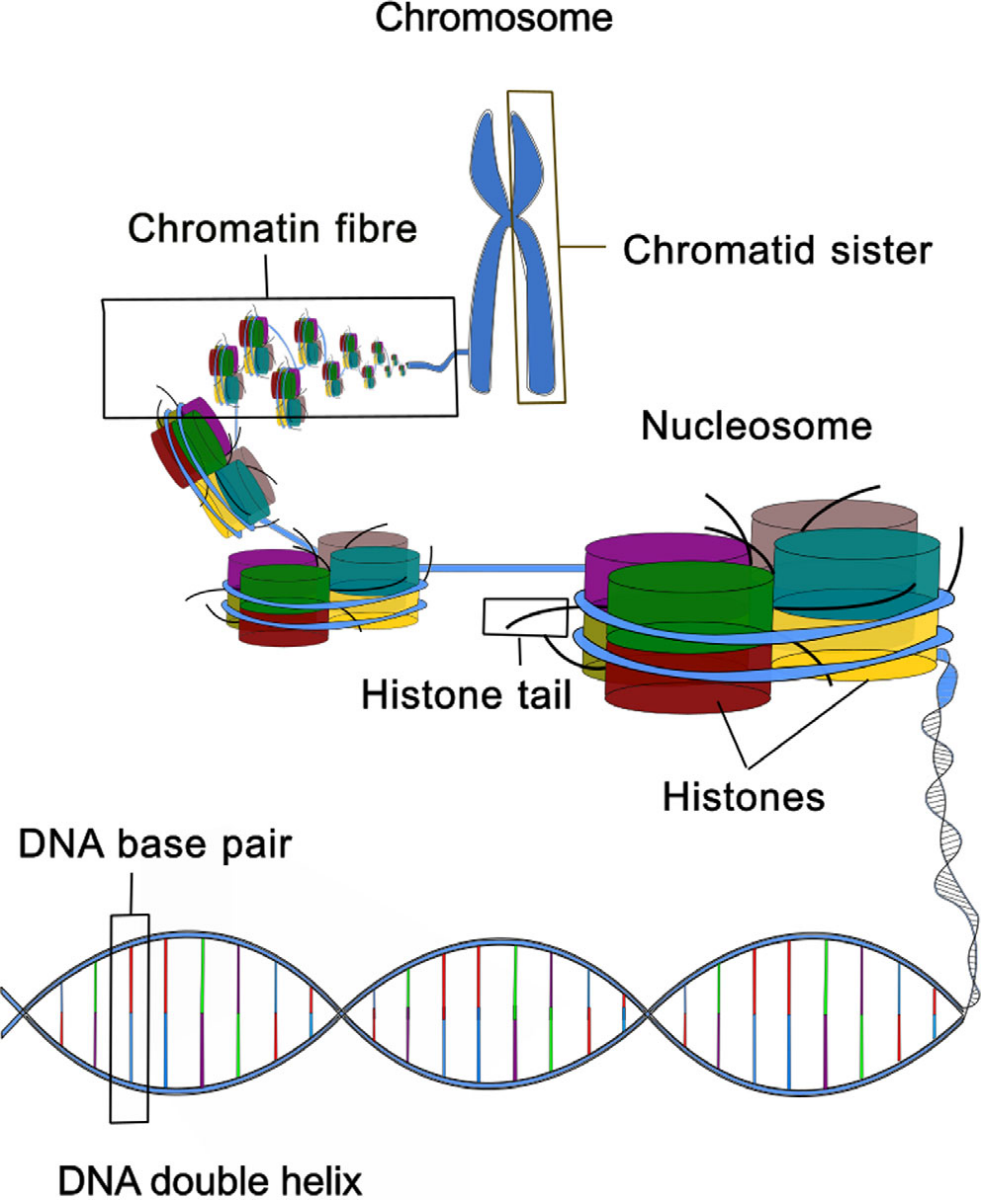
Structure of the chromosome. The smallest functional unit of DNA is the base pair. Approximately 147 base pairs wrap around eight histones to form the nucleosome, the basic unit of chromatin. The nucleosome structure is tightly organised to create the chromatin fibre, which forms the chromatid. The chromatid is duplicated, making two identical copies of the genetic sequence. These two identical chromatids together form a chromosome.

Acting alongside the nuclear lamina structure is chromatin. Chromatin is a multi‐level structure (Fig. 2) which at its smallest scale is DNA, with approximately 147 DNA base pairs wrapping around a core of eight histones to form a nucleosome. Nucleosomes arrange together to form chromatin fibres (Hou *et al*., 2023; Zhou, Gaullier & Luger, 2019), with the chromatin fibre ultimately forming the chromosome (Antonin & Neumann, 2016). Chromatin is a highly dynamic structure, able to condense and open to allow access to DNA (Antonin & Neumann, 2016; Grubert *et al*., 2020; Kadauke & Blobel, 2009; Martin & Cardoso, 2010; Segert, Gisselbrecht & Bulyk, 2021), representing an important aspect of epigenetic regulation. The different states of chromatin result in varying mechanical properties, with open chromatin more compliant whilst condensed chromatin is stiffer (Nava *et al*., 2020). The importance of lamins and chromatin in determining nuclear mechanics (Stephens *et al*., 2017) makes them principal considerations herein.

A role of lamins within cancer has been considered from a genetic perspective, but with inconsistent conclusions as the upregulation of lamin A/C gene expression can be associated with better [e.g. ovarian (Wang *et al*., 2019), prostate and breast cancer (Reis‐Sobreiro *et al*., 2018)] or worse [e.g. lung cancer (Roncato *et al*., 2021; Zhang *et al*., 2018)] outcomes (Dubik & Mai, 2020). Similar contradictions can be seen for lamin A/C protein expression, with up‐ or down‐regulation having varying effects on prognosis within different cancers (Bell *et al*., 2022; Kong *et al*., 2012). Thus, despite a prominent role in nuclear mechanics, there is a lack of clarity on the role of lamin A/C protein and gene expression within cancer. Yet, this variability in the role of lamin A/C within cancer could point to the significance of mechanics. Roncato *et al*. (2021) showed that increased metastasis associated with lamin A/C was due to improved cell survival, increasing metastatic potential. Inhibiting lamin A/C has been shown to result in greater DNA damage when migrating through small pore sizes (Wang *et al*., 2019) as the increased compressive forces exceeded the cell's stiffness. This indicates that weakening of the cell nucleus reduces cell survival. By contrast, Reis‐Sobreiro *et al*. (2018) found that loss of lamin A/C led to greater invasiveness, aggressiveness, and slightly increased metastatic potential. Thus, these results all support a critical role for the structural integrity of the cell: higher stability can reduce aggressiveness and metastasis but improve cell survival, whilst lower stiffness reduces cell survival but increases metastatic potential. Given this, future studies should investigate the changes in lamina structure.

Xu *et al*. (2022) investigated several different mouse and human cancerous and non‐cancerous cells, examining healthy, pre‐cancerous, and cancerous cells in each case. Using high‐resolution imaging, they detailed the progression of lamin A/C and chromatin structural breakdown. In healthy cells, the lamin A/C perimeter is intact and distinct from chromatin. This is compromised in pre‐cancerous cells, with colocalization of lamin A/C and chromatin, changes in cell shape, and erosion of the lamin A/C perimeter. These changes are further pronounced in cancerous cells, with extensive colocalization and lamin A/C erosion from the nuclear periphery. These findings appear preserved across different cell lines used within the study and are consistent with previous findings showing nuclear shape change in cancer cells (Singh & Lele, 2022; Tocco *et al*., 2018). Cumulatively, the structural findings of Xu *et al*. (2022) and genetic investigations (Roncato *et al*., 2021; Wang *et al*., 2019) support lamin A/C as an important contributor to nuclear mechanics.

The compromised nuclear stiffness hypothesised to be the driving mechanical event within cancer (Fischer *et al*., 2020; Massey *et al*., 2024) helps contextualise the role of lamin A/C within cancer. Increasing lamin A/C protein levels may partially restore nuclear stiffness. As loss of nuclear stiffness predicts cancer aggressiveness (Deville & Cordes, 2019; Fischer *et al*., 2020), improving nuclear stiffness may inhibit cancer progression. However, this increased stiffness also improves cell resilience (Roncato *et al*., 2021) and therefore cell survival, meaning metastasising cells are more likely to succeed. Conversely, reductions in lamin A/C will increase cancer progression as the nucleus is further destabilised, yet cell survival is compromised (Reis‐Sobreiro *et al*., 2018). Thus, the results indicate that nuclear structure is the determining factor, yet the variable results of both increased and decreased lamin A/C protein provide some indication they are not the primary driver of altered nuclear mechanics within cancer. Zhang *et al*. (2018) provide an additional insight to lamin A/C and nuclear mechanics. They showed that knockdown of Nestin within several cell lines (H1299, A549, LN229, C4‐2, PC‐3, HEK293T, U251, TE‐1, and Eca‐109) resulted in altered regulation of lamin A/C, causing the removal of lamin A/C from the nucleus and its eventual breakdown. This loss of lamin A/C resulted in cell senescence. Nestin is a type VI intermediate filament and therefore contributes to the mechanics of the cell cytoskeleton (Lendahl, Zimmerman & McKay, 1990). However, previous research has shown that Nestin is associated with increased cancer cell migration and metastasis (Ishiwata, Matsuda & Naito, 2011; Szymanska‐Chabowska *et al*., 2021). The apparent contradiction between the structural genesis hypothesis presented here and a structural protein within the cell being associated with increased malignancy is resolved by the findings of Yamagishi *et al*. (2019). Using a mouse cancer cell line (FP10SC2), they observed that Nestin inhibited the interaction of other structural proteins within the cell, leading to diminished cell stiffness. Thus, cell and nuclear mechanics are more than the sum of their parts, and consideration of nuclear mechanics requires identification of the principal mechanical driver or drivers. Here, the findings of Zhang *et al*. (2018) are useful, as the induced senescence following lamin A/C depletion provides potential insight into the role of lamin A/C within nuclear stiffness mediation. Mitosis is dependent upon chromatin condensation (to ~39%; Martin & Cardoso, 2010), with chromatin condensation partly dependent upon histone modifications (Antonin & Neumann, 2016), which are partly regulated by lamin A/C (Dechat *et al*., 2008). Thus, loss of lamin A/C eventually may compromise chromatin architecture (Xu *et al*., 2022) sufficiently to prevent the necessary condensation and therefore arrest cell reproduction. Indeed, Zhang *et al*. (2018) provide evidence that loss of condensed chromatin was universally observed across lamin A/C‐deficient cells. With subsequent overexpression of lamin A/C able to restore cell properties, and other studies establishing lamin A/C as an important regulator of chromatin architecture (Shimi *et al*., 2008), and chromatin architecture an important mediator of nuclear mechanics (Nava *et al*., 2020), the results of Zhang *et al*. (2018) make the importance of lamin A/C in nuclear mechanics clear. However, the inconsistent presentation of lamin A/C dysregulation provides evidence that the contributions of lamin A/C to the progression of cancer are secondary. Further, with the senescence observed by Zhang *et al*. (2018) possibly having its ultimate origin in chromatin architecture changes, as suggested by the need for chromatin condensation in mitosis (Martin & Cardoso, 2010), the results suggest that lamin A/C's effects may be due to its influence on chromatin structure. The data would suggest that lamin A/C regulates nuclear mechanics and mechanically dependent functions, but that this regulation depends upon its mediation of chromatin structure. Thus, chromatin is implicated as the principal determinant of cell nucleus mechanics, leading to a focus on the epigenetic structure within cells.

Chromatin is the structure that emerges from DNA and histone binding (Fig. 2) (Hou *et al*., 2023; Zhou *et al*., 2019), and as such is dependent upon these constituent materials and the basic structure they create, the nucleosome. This emergent structure thereby is key to chromatin structure and mechanics, making the interaction between DNA and histones a cornerstone of nuclear mechanics. The studies of Xu *et al*. (2020) and Xu *et al*. (2022) revealed a progressive breakdown of heterochromatin (condensed chromatin), smaller heterochromatin clusters, and reduction in nuclear radius from healthy to cancerous cells taken from mice. Healthy and pathological cells from neoplasia patients were also compared, with findings again showing a breakdown in heterochromatin, reduced cluster sizes, and a reduction in nuclear radius size. The results led the authors to suggest that early carcinogenesis involves the decompaction of heterochromatin and compromise of the wider chromatin architecture, resulting in increased transcription activity and leading to genomic instability. Thus, they argue that dysregulated nucleosome structure is a mechanistic component of the hallmarks of cancer. Certainly, the results support that both the epigenetic and transcriptional changes observed in cancer can have a structural origin.

Further support comes from the Barr body, the inactive X‐chromosome in females (Carone & Lawrence, 2013). It is characterised by highly condensed heterochromatin (Smeets *et al*., 2014), indicating its inactivity is derived from being architecturally inaccessible to genetic processes (Giri & Prasanth, 2012; Martin & Cardoso, 2010; Ramos‐Alonso *et al*., 2023). Within breast cancers, Barr bodies occur far less frequently compared to healthy controls (Chaligne *et al*., 2015). Loss of Barr bodies has been linked to more aggressive tumours (Richardson *et al*., 2006; Rosen *et al*., 1977; Smethurst *et al*., 1981), and it has been suggested that overexpression of specific X‐linked genes contributes to tumour formation (Jazaeri *et al*., 2002; Richardson *et al*., 2006). These results, in conjunction with those of Xu *et al*. (2020) and Xu *et al*. (2022), provide a reasonable basis for viewing decondensed heterochromatin as both a marker of cancer and predictor of cancer aggressiveness. The loss of heterochromatin points to a weakening of the nucleosome and indicates that compromised associations between DNA and histones are producing the loss of nuclear stiffness (Santos & Toseland, 2021; Shimamoto *et al*., 2017; Strickfaden *et al*., 2020). This holds significant implications for the entirety of the genetic landscape.

### (3) Genetics, epigenetics and mechanics

Chang, Shen & Yan (2021) detail findings that contextualise the implications of chromatin structure breakdown. Using *Drosophila* cells, they investigated the effects of high dietary sugar intake and chromatin structure on cancer outcomes. Two *Drosophila* models were used to achieve this: Ras/Src, which are proteins involved in cell signalling pathways (Benard, Naor & Seger, 2001), and Scrib, a membrane protein mediating cell migration and proliferation (Humbert, Russell & Richardson, 2003). The interaction between Ras/Src‐activated (Enomoto, Takemoto & Igaki, 2021) or Ras‐activated and Scrib‐deficient cells (Wu, Pastor‐Pareja & Xu, 2010) can drive malignancy and tumour invasion (Enomoto, Takemoto & Igaki, 2021; Wu, Pastor‐Pareja & Xu, 2010). Results showed that high dietary sugar intake‐induced tumours had reduced heterochromatin within cancerous cells. Increasing heterochromatin formation led to reduced lethality, tumour formation, proliferation, and genome instability whilst increasing apoptosis. Rose *et al*. (2022) used mouse neural cells to examine nuclear structure alterations preceding apoptosis. They demonstrated that nuclear condensation to approximately 26% its original size was a necessary structural event that precedes apoptosis initiation. Failure to condense results in significant reductions in apoptosis, a hallmark of cancer (Hanahan & Weinberg, 2011). The significance of nuclear condensation in apoptosis resistance of cancer cells is further supported by Cheng *et al*. (2009). Mammary carcinoma cells (67NR and EMT6) were seeded onto an *in vitro* constrained, micro‐beaded environment. As the cell population grew, they generated increasing compressive forces and restoration of apoptosis was strongly related to increased compressive stress. Although the nuclear structure was not investigated, these results are consistent with nuclear compression as a necessary precursor to apoptosis. Furthermore, restoration of apoptosis in cancer cells subjected to compression indicates the apoptotic pathway is intact and is indeed mechanically constrained.

Similarly to apoptosis, mitosis also requires nuclear condensation, although not as severe (Martin & Cardoso, 2010; Rose *et al*., 2022) and derived through different compressive mechanisms (Antonin & Neumann, 2016; Hudson *et al*., 2003; Phengchat *et al*., 2016; Rose *et al*., 2022). The relevance here is the complex architectural changes necessary to allow proper chromatin compression during mitosis (MacGregor, Adams & Gilbert, 2019). These architectural changes are dependent upon significant histone epigenetic regulation (Andres *et al*., 2020), important as compromised chromatin structure indicates the interaction between DNA and histones may be compromised, weakening the nucleosome. Compromised nucleosome stability is consistent with the high rate of cancer‐associated histone mutations (Bagert *et al*., 2021). Using humanised yeast cells and whole‐genome sequencing data mining, Bagert *et al*. (2021) investigated histone mutations, showing they upregulated cancer‐associated gene pathways in mammalian cells. *In vitro* reconstituted nucleosomes and HeLa nucleosomes also were investigated (Arimura *et al*., 2018). It was found that the Glu76 mutation common within cancer (Arimura *et al*., 2018) distorts the nucleosome structure by inhibiting the interaction of H2B and H3–H4, ultimately destabilising the nucleosome. Accompanying this instability was a greater rate of colony growth in the mutated histone HeLa cell variants, indicating oncogenic‐like transformation through reduced contact inhibition leading to greater colony formation ability. Testing of additional histone mutations (H3 E97K and H2A.Z.1) found nucleosome instability to characterise the mutations with varying effects on colony formation. Indeed, the evidence for histone dysfunction and mutation within cancer progression is rapidly growing (Nacev *et al*., 2019; Pereira *et al*., 2023). Cumulatively, the results support nucleosome mechanics as an important factor, yet the varying frequency of histone modifications within different cancers (Nacev *et al*., 2019; Pereira *et al*., 2023) highlights that histones alone do not provide an answer to the changing mechanical state.

Despite the variable effects of histone mutations, the role of histones increases in importance when considered within the wider chromatin structure. Ishii *et al*. (2008), using both HeLa cell and animal cell *in vitro* models, showed that histone deacetylation 3 (HDAC3) is a crucial regulator of mitotic segregation accuracy through its mediation of chromatin condensation. Knockdown of HDAC3 resulted in severe chromosomal dysregulation due to poor microtubule–kinetochore attachments. The prevalence of dysregulated microtubule–kinetochore attachments within cancer cells (Bufalo & Degrassi, 2015; Hanahan & Weinberg, 2011; Herman *et al*., 2015) links histone modifications to common features of cancer. Specifically, the results of Ishii *et al*. (2008) indicate that chromatin condensation, as mediated by HDAC3, is fundamental to mitotic accuracy. Therefore, the compromised chromatin condensation observed within cancer (Gopi & Kidder, 2021; Gurrion, Uriostegui & Zurita, 2017; Rafique *et al*., 2015; Xu *et al*., 2020; Xu *et al*., 2022) threatens mitotic accuracy and can thereby generate chromosomal instability. The high prevalence of aneuploidy and chromosomal instability within cancer – from 26% (thyroid carcinomas) to 99% (glioblastomas, testicular germ cell tumours) (Taylor *et al*., 2018) – demonstrates this is a significant factor within disease progression. However, the ability for cancer to form without such genetic instabilities suggests they are a symptom rather than a cause; their high frequency indicates that these instabilities are closely linked to mechanistic drivers of cancer.

What emerges from the histone data is that, rather than histones specifically, it is their effect on nucleosome structure that is of greater interest. This is highlighted by the results of Arimura *et al*. (2018) and Ishii *et al*. (2008), with the effects of histones occurring through alterations to chromatin architecture, a process dependent upon nucleosome structure. Therefore, histones are part of a larger structure and cannot be considered in isolation. Emphasising the importance of the whole nucleosome structure, Strickfaden *et al*. (2020) used isolated nuclei from mouse embryonic fibroblasts (C3H/10T1/2) to demonstrate that the mechanical behaviour of nuclei is dependent upon chromatin condensation. Decondensed, open chromatin behaves like a fluid whereas condensed chromatin functioned like a solid. This was supported by the results of Herman *et al*. (2020). Therefore, the altered mechanical behaviour of cancer nuclei, becoming more fluid like (Fuhs *et al*., 2022), is consistent with losses in chromatin density. But further to this, it indicates that cells can dynamically alter their nuclear mechanics through modulating the state of chromatin. This, together with the consistently reduced nuclear stiffness across cancers (Fischer *et al*., 2020; Massey *et al*., 2024; Xu *et al*., 2012; Xu *et al*., 2022), provides evidence that compromised chromatin condensation is key. With the nucleosome being the structural unit mediating chromatin density, these findings indicate that nucleosome structure is compromised within cancer cells. Thus, the whole nucleosome, through mediation of chromatin state, is implicated in cancer cell mechanics. Specifically, the loss in condensed chromatin (Gopi & Kidder, 2021; Gurrion *et al*., 2017; Rafique *et al*., 2015; Xu *et al*., 2020; Xu *et al*., 2022) indicates that nucleosomes are weakened, and close associations between their constitutive components (DNA and histones) are compromised.

In this way, structural destabilisation of the nucleus can result in genomic instability, providing a potential mechanical pathway to the cause of cancer. With nuclear destabilisation possibly a unifying characteristic across all cancers (Fischer *et al*., 2020; Massey *et al*., 2024; Xu *et al*., 2020; Xu *et al*., 2022), and predictive of cancer aggressiveness and metastatic potential (Fischer *et al*., 2020), this provides a foundation for a mechanical genesis of cancer. This mechanical perspective originates from compromised nucleosome mechanics and details a potential pathway for mechanical dysregulation of the cell nucleus that can result in severe, extensive, and varied genetic mutations as a direct consequence of mechanical destabilisation.

## IV. IMPLICATIONS AND DIRECTIONS FOR FUTURE RESEARCH

The conclusions presented herein are a departure from the traditional perspective of cancer, in which genetic mutations drive the pathology. Although the authors understand and acknowledge that such genetic events are a crucial component in pathology progression, the conclusions presented here suggest that this genetic landscape is the symptom of an upstream mechanical event. Indeed, the changes observed at the genetic, epigenetic, and tissue level are argued to be the direct result of this mechanical compromise.

The repercussions of a mechanical origin to cancer hold particular interest in the development of treatments. Traditional methods, such as chemotherapy and radiotherapy, function by destroying the cancerous cells, although their non‐specific nature means such effects occur also in healthy cells (Hubenak *et al*., 2014; Rebe & Ghiringhellie, 2015). However, in contrast to the antagonistic nature of such treatments, the hypothesis presented here suggests treatments to *benefit* the mechanical properties of cancer cells may hold promise. If the cell stiffness of cancer cells can be restored, then the potential driver of tumour formation may be removed and allow deconstruction of the tumour. Further, it may lead to genetic stability, with increases to aneuploidy or further mutations being prevented. Thus, cancer becomes a static target whilst deconstruction of the tumour improves immune system access. Finally, by restoring cell mechanical properties, the apoptotic pathway could possibly be restored, allowing greatly increased rates of programmed cell death. Therefore, what emerges from the theory presented here is that, rather than being an invading force, cancer is a compromised cell that requires support to return to normal function.

The new treatment avenues that emerge represent an important topic for future research. Yet, despite their importance, such endeavours will require greater foundations than currently exist. Three primary avenues exist for future research prior to exploring such treatment avenues. The first is the interaction between cell stiffness and tumour stiffness. If the tumour functions as a structural scaffold, it would be expected that reducing cell nuclear stiffness will result in greater tumour stiffness as an attempt to compensate. The second is the interaction of compromised cell mechanics and the genetic landscape within cancer cells. Research that explores genetic mutations would benefit from describing the local chromatin structure of mutated genes. Further, how the extent of chromatin unravelling impacts aneuploidy and genetic mutations more generally may give greater insight into any role of mechanical changes inside the nucleus in driving the genetic landscape of cancer. Thirdly, mechanisms that compromise nucleosome integrity need to be explored. Whether any mechanisms that emerge from such investigations are known to occur in carcinogens may represent some of the first steps into treatment investigations.

## V. CONCLUSIONS

(1) This review presents the hypothesis that cancer is a mechanically initiated pathology. We suggest a potential pathway from compromised chromatin structure to genetic dysregulation at both the genetic and epigenetic level, with aneuploidy possible due to mitotic errors. Further, mechanical involvement in apoptosis indicates how nuclear mechanics can influence regulatory processes that could otherwise destroy the cancerous cell. Finally, the formation of tumours within cancer is argued to be a means of increasing cell stiffness to restore mechanical homeostasis. Therefore, aspects of cancer pathology may be a direct cellular response to improving its mechanical properties, suggesting that restoration of cell stiffness may lead to marked improvement in key components of cancer pathology.
(2) Supplementing the primary hypothesis of mechanical origins to cancer is the hypothesis of cell mechanical homeostasis. Contrasting with the traditional mechano‐transduction model of external forces acting upon the cell through deformation, we hypothesise that the cell operates within a target stiffness range. Actions that move the cell from this range will elicit a cellular response. Thus, mechano‐transduction becomes a contributing factor to a larger mechanical environment. Within the compromised cancer cell, the interaction with external mechanical cues is severely compromised as the reduced cell stiffness dampens any external events.
(3) This hypothesis provides a new avenue for investigation of cancer. Specifically, nucleosome degradation as the trigger for nuclear destabilisation represents a specific target for investigation. Further, it suggests the genetic landscape within a cancer may be a symptom of upstream mechanical events. Thus, whilst able to influence the manner and rate of cancer progression, the genetic landscape does not determine its initiation. Therefore, the conserved mechanics of cancer, and its ability to affect a diversity of genetic and epigenetic processes become a point of great importance for future research.
